# *Trypanosoma cruzi* Secreted Cyclophilin *Tc*CyP19 as an Early Marker for Trypanocidal Treatment Efficiency

**DOI:** 10.3390/ijms241511875

**Published:** 2023-07-25

**Authors:** Alina E. Perrone, Mariana Pinillo, Marcela S. Rial, Marisa Fernández, Natalia Milduberger, Carolina González, Patricia L. Bustos, Laura E. Fichera, Susana A. Laucella, María Cecilia Albareda, Jacqueline Bua

**Affiliations:** 1 Instituto Nacional de Parasitología Dr. Mario Fatala Chaben—ANLIS-Malbrán, Av. Paseo Colón 568, Buenos Aires 1063, Argentina; alinaperrone@yahoo.com.ar (A.E.P.); marianapinillo2017@gmail.com (M.P.); marcelarial2@hotmail.com (M.S.R.); marisa.fernandez@gmail.com (M.F.); nmilduberger@gmail.com (N.M.); caro.gonzalez327@gmail.com (C.G.); pato54mar@yahoo.com.ar (P.L.B.); lfichera@yahoo.com (L.E.F.); slaucella@yahoo.com (S.A.L.); mcalbareda@gmail.com (M.C.A.); 2 Consejo Nacional de Investigaciones Científicas y Técnicas, Buenos Aires 1063, Argentina

**Keywords:** Chagas, *Trypanosoma cruzi*, cyclophilin, *Tc*CyP19, ELISA, biomarker, benznidazole, nifurtimox, parasiticidal treatment

## Abstract

Cyclophilins (CyPs) are a family of enzymes involved in protein folding. *Trypanosoma cruzi*, the causative agent of Chagas disease, has a 19-kDa cyclophilin, *Tc*CyP19, that was found to be secreted in parasite stages of the CL Brener clone and recognized by sera from *T. cruzi*-infected mice and patients. The levels of specific antibodies against *Tc*CyP19 in *T. cruzi*-infected mice and subjects before and after drug treatment were measured by an in-house enzyme linked immunosorbent assay (ELISA). Mice in the acute and chronic phase of infection, with successful trypanocidal treatments, showed significantly lower anti-*Tc*CyP19 antibody levels than untreated mice. In children and adults chronically infected with *T. cruzi*, a significant decrease in the anti-*Tc*CyP19 titers was observed after 12 months of etiological treatment. This decrease was maintained in adult chronic patients followed-up 30–38 months post-treatment. These results encourage further studies on *Tc*CyP19 as an early biomarker of trypanocidal treatment efficiency.

## 1. Introduction

Chagas disease, which is produced by the unicellular hemoflagellate parasite *Trypanosoma cruzi*, is one of the most neglected tropical diseases. It can be transmitted by triatomine insects by vertical transmission and, to a lesser extent, by blood transfusions and food contamination [1]. This disease affects about six million people, 12,000 of whom die each year. The highest prevalence of Chagas disease in the Latin American region is found in Bolivia (6.75%), Argentina (4.13%) and Paraguay (2.54%) [2]. This infection is also spread by migration of infected people to non-endemic areas such as the USA, Canada, many European countries and the Western Pacific [3]. The course of Chagas disease consists of three distinct phases: acute, indeterminate and chronic. The initial acute phase occurs after the entry of *T. cruzi* into the host. At this stage, death occurs in a few cases (<5–10% of symptomatic cases) as a complication of acute myocarditis and/or meningoencephalitis. After the acute phase, the infection evolves to a chronic phase. In the chronic phase, approximately 60–70% of patients never present apparent clinical manifestations of the disease, whereas the remaining 30–40% develop the cardiac and/or gastrointestinal form of Chagas disease [4]. This disease is very complex and the persistence of the parasite in tissues has implications for the development of clinical manifestations [5].

Two of the licensed drugs with proven efficacy against Chagas disease are Benznidazole (BNZ) and Nifurtimox. Trypanocidal treatment with these drugs in both adults and children is effective in terms of seroconversion and parasite load clearance [6,7]. Current guidelines recommend parasiticidal treatment in the acute phase of *T. cruzi* infection, in children younger than 18 years old, in women of childbearing age, in patients with reactivated parasite infection after immunosuppression and in chronic patients with mild or no cardiac alterations [8]. Treatment in the chronic phase of the infection in patients with severe cardiomyopathy exerts a trypanocidal effect in certain geographical areas, but does not lead to an improvement in the clinical outcome [9]. BNZ or nifurtimox treatments are better tolerated by infants than by older children or adult patients, who frequently present side effects, dermatitis by hypersensitivity and digestive intolerance due to a nitro-heterocyclic-compound-related mechanism of action. In fact, about 12–18% of patients suspend treatments due to these side effects [10,11,12].

One of the methods currently used to assess treatment efficiency is seroconversion, and follow-up studies have demonstrated that the rate of reversion to negative serology is very high when *T. cruzi*-infected babies are treated [13]. The decrease in anti-parasite antibodies is expected to be slower than in babies when treatments are administered between 5 and 14–16 years of age [14,15], but faster than treated adults, since serology in adults might take years or even more than a decade to detect a decrease in specific antibody levels [16].

Preclinical studies in a murine model aiming to obtain a more efficient parasiticidal treatment with less undesirable effects showed that lower BNZ concentrations alone or in combination with Allopurinol (ALLO), and even other formulations such as nano- or micro-particles, improved the solubility and biopharmaceutical performance of the drug [17,18,19,20].

Previous studies have shown that *T. cruzi* overexpresses cyclophilins (CyPs), which are a family of proteins highly conserved among species. These proteins have peptidyl prolyl *cis-trans* isomerase (PPIase) activity involved in protein folding [21] and are inhibited by Cyclosporin A, an immunosuppressive agent [22]. In mammals, the most represented cyclophilin is CyPA, a cytosolic and secreted protein with many biological functions [23]. In our laboratory, we have described the CyP family of *T. cruzi*, composed of 15 coding genes [24]. When we analyzed the expression of these genes in the epimastigote stage, we were able to isolate four Cyclosporin A affinity proteins, identified by mass spectrometry as *Tc*CyP19, *Tc*CyP22, *Tc*CyP28 and *Tc*CyP40 with molecular weights of 19, 22, 28 and 40 kDa, respectively [24]. We further studied cytosolic *Tc*CyP19, which is homologous to mammalian CyPA, and found that it is abundantly expressed in epimastigotes, amastigotes and trypomastigotes and that it exhibits PPIase activity, sensitive to the inhibitory action of Cyclosporin A and its non-immunosuppressive derivatives [25,26,27]. Many cytosolic proteins secreted by *T. cruzi* have been described as virulence factors with immunostimulatory properties, and, when the parasite secretome was characterized, *Tc*CyP19 was found to be secreted [28]. This cyclophilin promotes epimastigote survival by neutralizing parasiticidal peptides in the reduviid *T. cruzi* vector [29]; when secreted by amastigotes, *Tc*CyP19 induces intracellular production of reactive oxygen species in host cells, promoting parasite growth [30], and is involved in infectivity and virulence [31].

The aim of this work was to analyze the antibody levels against *Tc*CyP19 in sera from *T. cruzi*-infected experimental animals and patients before and after treatment with trypanocidal drugs to evaluate its potential value as an early marker for the efficacy of trypanocidal treatment.

## 2. Results

### 2.1. TcCyP19 Cyclophilin Is Secreted in the Extracellular Environment

**Figure 1:**
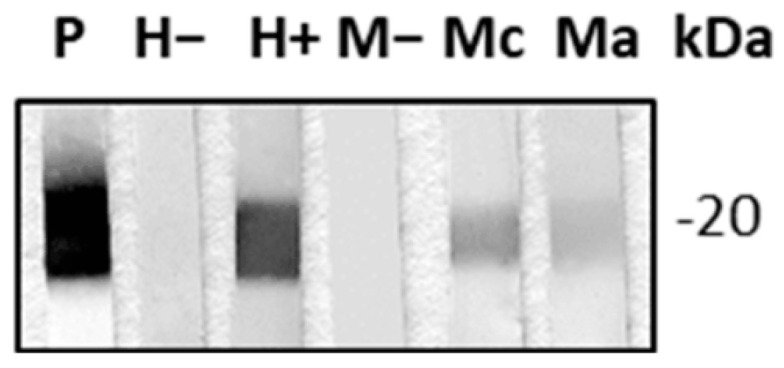
The *T. cruzi* recombinant protein, *Tc*CyP19, electrotransferred onto nitrocellulose strips, was recognized by sera from a chronically *T. cruzi*-infected patient (H+), a chronically *T. cruzi*-infected mouse (Mc) and an acute *T. cruzi*-infected mouse (Ma). As negative controls, sera from an uninfected human (H−) and an uninfected mouse (M−) were used. Polyclonal mouse antibodies against the *Tc*CyP19 recombinant protein were used as a positive control (P).

Since *Tc*CyP19 has been previously found to be excreted/secreted in different *T. cruzi* strains [29,30,31], we first analyzed the secretion of *Tc*Cyp19 in the *T. cruzi* CL Brener clone, the reference strain of the *T. cruzi* genome project, and the parasite source of this cloned gene. *Tc*CyP19 was found to be secreted in the supernatants of epimastigotes, trypomastigotes and amastigotes of cultures of the CL Brener clone. We then searched whether this protein elicits specific antibodies in *T. cruzi*-infected mice and humans. Anti-*Tc*CyP19 antibodies were found in the sera of mice infected with the *T. cruzi* Nicaragua isolate in the acute and chronic phase, as well as in the blood samples obtained from chronically *T. cruzi*-infected humans (Figure 1).

To determine whether this qualitative binding of antibodies against *Tc*CyP19 observed in *T. cruzi-*infected hosts was correlated to parasite levels of this secreted protein, a further analysis would be the quantification of specific antibodies when mice and humans diminish their parasitemia through a parasiticidal treatment. We then developed an enzyme-linked immunosorbent assay (ELISA) using *Tc*CyP19 recombinant protein as antigen.

### 2.2. Detection of Antibodies against TcCyP19 Protein in T. cruzi-Infected Mice

In our in-house ELISA to detect *Tc*CyP19, the sera from all uninfected mice were negative, with optical density (OD) values at 490 nm below 0.05. In contrast, all *T. cruzi*-infected mice developed OD values at 490 nm above 0.05, indicating a signal-to-cutoff ratio higher than 1, which indicates the presence of antibodies against the *Tc*CyP19 recombinant protein.

C3H/HeN mice inoculated with 1000 trypomastigotes of the *T. cruzi* Nicaragua isolate (*Tc*N) were treated in the acute phase of the infection with low doses of BNZ formulated in nanoparticles (10 and 50 mg/kg/day of BNZnps (BNZnp10 and BNZnp50)) [18].

**Figure 2:**
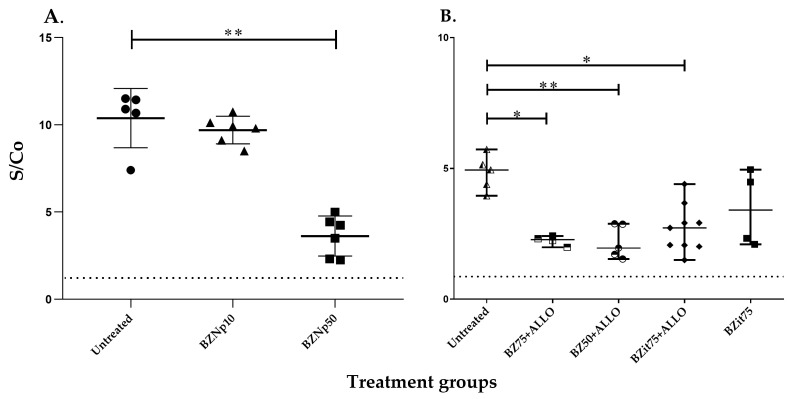
Anti-*Tc*CyP19 specific antibody levels tested in an in-house ELISA. (**A**) Serum samples were obtained from C3H/HeN mice infected with a *T. cruzi* Nicaragua isolate (*Tc*N) treated in the acute phase of the infection with a formulation of Benznidazole in nanoparticles. BNZnp10: mice treated with 10 mg BNZ/kg/day (▲); BNZnp50: mice treated with 50 mg BNZ/kg/day (■) (as described in Section 4.4) Sera from untreated *T. cruzi-*infected mice (●) were used as a positive control (** *p* < 0.01). (**B**) Serum samples were obtained from C57BL/6J mice infected with *Tc*N treated in the chronic phase of the infection. Mice were treated with continuous doses of BNZc75+ALLO (□) and BNZc50+ALLO (ο) or intermittent treatments with one dose of BNZit75+ALLO (♦) or BNZit75 (■) (as described in Section 4.5). Sera from untreated *T. cruzi-*infected mice were used as positive control (△) (** *p* < 0.01, * *p* < 0.05). Results are expressed using the signal-to-cutoff (S/Co) ratio, by dividing the OD value of the samples tested by the OD value of the assay cut-off. The dash line represents S/Co = 1, the ratio obtained with sera from uninfected mice.

We then searched for anti-*Tc*CyP19 antibodies in sera from these treated mice by using our in-house ELISA. Mice treated with BNZnp50 showed a significant decrease in antibody levels compared to untreated mice in the acute phase of the infection. In contrast, mice treated with BNZnp10 showed high titers of anti-*Tc*CyP19 antibodies, correlated with an inefficient parasiticidal treatment (Figure 2A). Since untreated *Tc*N-infected C3H/HeN mice resulted only in a 15% survival rate, with the aim to study drug treatments during the chronic phase of the *T. cruzi* infection, another mouse model was assayed: C57BL/6J mice inoculated with 3000 *Tc*N trypomastigotes. These mice showed a survival rate of 45% after the acute phase. We then evaluated the levels of anti-*Tc*CyP19 antibodies in mice in the chronic phase of *T. cruzi* infection by comparing the administration of continuous and intermittent treatments [19]. The levels of anti-*Tc*CyP19 antibodies in mice treated with continuous administration of BNZ and in those treated with intermittent treatment (BNZit75) in combination with ALLO were lower and showed a significant difference with antibody titers of untreated *T. cruzi*-infected mice. However, mice receiving BNZit75 without the addition of ALLO did not exhibit significantly different levels of anti-*Tc*CyP19 antibodies compared to those of untreated mice (Figure 2B).

### 2.3. Detection of Antibodies against TcCyP19 Protein in Chronically Infected T. cruzi Patients

Sixteen *T. cruzi*-infected adults and seventeen *T. cruzi*-infected children who had received trypanocidal treatment were evaluated in this study. The data of recruited patients, including gender, age, drug treatment and time at which the blood sample was obtained after the treatment, are summarized in Table 1. Adult and pediatric patients were treated with BNZ or Nifurtimox (as described in Section 4.6).

Human sera were tested with the in-house ELISA developed to detect *Tc*CyP19 antibodies. All serum samples from uninfected humans showed OD values below 0.05 at 490 nm. In contrast, most of the serum samples from *T. cruzi*-infected humans developed OD values above 0.05 with a signal-to-cutoff ratio higher than 1, which evidenced the presence of anti-*Tc*CyP19 antibodies.

In Table 1, “Children, group A” refers to those children who achieved seroconversion after treatment. “Children, group B” refers to those who sustained serological responses after a 5-year post-treatment follow-up [15].

From each recruited adult patient, we obtained one blood sample previous to treatment with BNZ or Nifurtimox, and another sample after the complete drug treatment. Anti-*Tc*CyP19 antibodies were tested by our in-house ELISA in these paired samples, before and after parasiticidal treatment in the first blood sample withdrawn after treatment at a mean of 11.9 months for adults and 6 months for children. 

**Figure 3:**
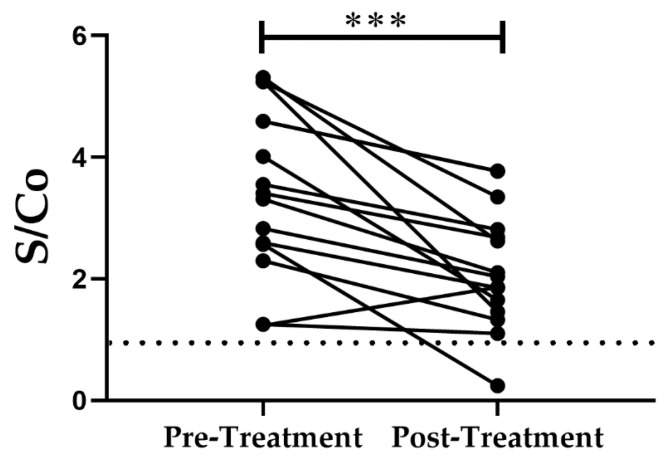
Comparison of samples from adult treated patients withdrawn before and after parasiticidal treatment. Antibody levels against *Tc*CyP19 recombinant protein detected in sera from treated patients significantly decreased after treatment. Blood samples after treatment were obtained at a mean of 11.9 months. A *p* value of 0.0005 (***) indicates a significant difference between the two sample groups using a parametric analysis. The dash line represents S/Co = 1, a ratio obtained with sera from uninfected patients.

The antibody levels against the *Tc*CyP19 recombinant protein from adult *T. cruzi*-infected and treated patients are shown in Figure 3. 

**Figure 4:**
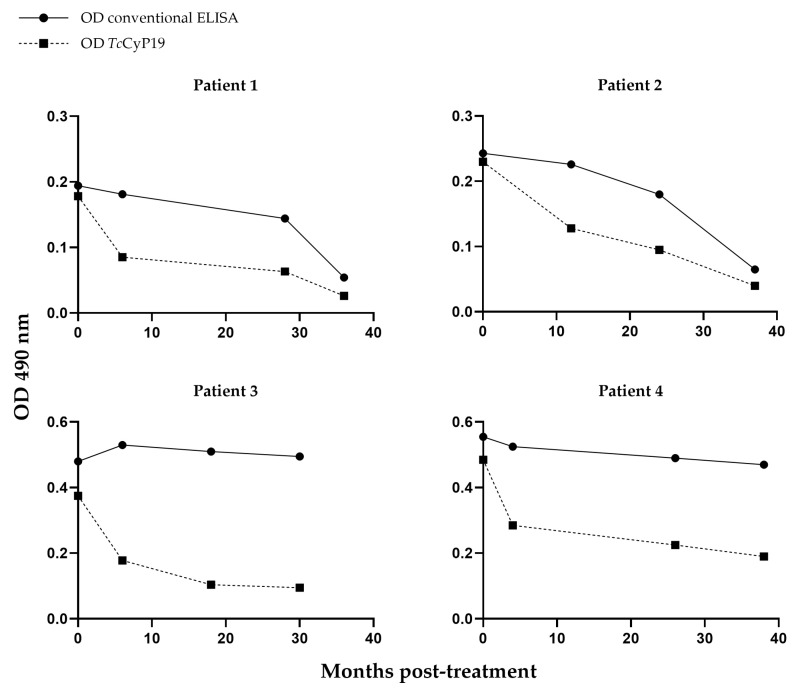
Anti-*Tc*CyP19 antibodies - - -■- - - and anti-*T. cruzi* antibodies -●- by conventional ELISA serology from patients treated with trypanocidal drugs as described in Section 4.8. Post-treatment blood samples obtained from Patient 1 at 4, 26 and 38 months; from Patient 2 at 6, 12, 24 and 37 months; from Patient 3 at 6, 18 and 30 months; and from Patient 4 at 6, 28 and 36 months. A decrease in the reactivity of this recombinant protein can be seen regarding the baseline reaction at 4–6 months after therapy. Results are expressed as OD value at 490 nm.

It is worth noting that the serum samples of *T. cruzi*-infected patients in the chronic phase showed great variability regarding the reactivity to this recombinant protein, a fact that can also be observed in the conventional ELISA method and in Figure 4. From the adult patients studied, we obtained three to four blood samples at different times, from 6 to 38 months, after treatment. The decrease in antibodies against *Tc*CyP19 recombinant protein and total *T. cruzi* antigens detected by the conventional ELISA assay can be visualized individually in Figure 4. In these four adult patients, the anti-*Tc*CyP19 antibody titers allowed us to visualize a successful trypanocidal effect earlier, between 4 and 6months post-treatment, compared with the antibody levels measured by the conventional ELISA serology (Figure 4).

**Figure 5:**
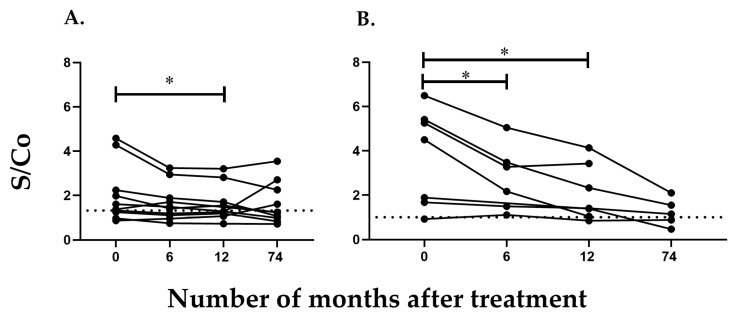
Anti-*Tc*CyP19 antibodies from children treated with trypanocidal drugs as described in Material and Methods. Post-treatment blood samples were withdrawn at 6, 12 and a mean of 74 months (range 48–120 months). (**A**): Group of children who achieved seroconversion by conventional serology after treatment. (**B**) Group of children who sustained serological responses after a 5 year post-treatment follow-up. * *p* < 0.05.

Serum samples from children treated with BNZ or Nifurtimox were grouped in two categories according to the results of the conventional serology after treatment at the end of follow-up: a group who achieved seronegativization after 12 months of trypanocidal treatment (group A), and in another group who did not achieve seronegativization (group B) [15]. The levels of antibodies against the *Tc*CyP19 recombinant protein found in both groups were significantly different (*p* = 0.0377 for group A and *p* = 0.0199 for group B) after one year of trypanocidal therapy (Figure 5). In addition, a significant decrease in anti-*Tc*CyP19 protein was also found 6 months post-treatment (*p* = 0.0296) in children of group B, who did not show a decrease in the conventional serological response (Figure 5).

## 3. Discussion

*Tc*CyP19 is highly expressed in all studied parasite stages. We found it secreted in the supernatants of parasite cultures in the *T. cruzi* CL Brener clone, the reference strain in the *T. cruzi* genome Project. *Tc*CyP19 sequence was deposited in GenBank with the Accession number AI021872. There is a large amount of evidence that Cyclophilin A (CyPA), its homologous protein in mammals, is secreted by different cell types [32]. Other research groups have previously demonstrated that *Tc*Cyp19 is secreted by epimastigotes of several *T. cruzi* strains [29]. This protein has also been found secreted by trypomastigotes of the *T. cruzi* Y strain, and in the host cell cytosol by intracellular amastigotes [31]. *Tc*CyP19 homologous protein has also been found in the secretome of African trypanosomes [33].

The amino acid sequence of *Tc*Cyp19 does not indicate a secretion signal, and in addition, in the absence of an endoplasmic reticulum (ER) signal sequence, neither *Tc*CyP19 nor CyPA are secreted through the classical ER-Golgi secretory pathway. More experimental evidence is needed to assess the secretion mechanism of these cyclophilins.

Biomarkers are defined as biological molecules found in blood or other body fluids that could be measurable indicators of a condition or disease. In this work, we analyzed a secreted *T. cruzi* protein to assess the efficiency of parasiticidal treatments in *T. cruzi*-infected hosts. When trypanocidal drugs reduce parasite loads in treated hosts, a logical consequence is the lower amount of parasite-secreted proteins [34].

We used the signal-to-cutoff (S/Co) ratio to express the results of our in-house ELISA—*Tc*CyP19—which has been very useful in the screening of viral infections such as hepatitis C because it accurately predicts HCV viremia [35,36] and allows the clinical classification of HIV-infected patients [37].

*T. cruzi* Nicaragua isolate-infected mice treated in the acute phase of infection with BNZ formulated in nanoparticles survived up to 60 days post-treatment, confirming trypanocidal efficiency, while 85% of untreated mice did not survive the *T. cruzi* infection. Mice treated with BNZnp50 in the acute phase of the infection, which was the most efficient treatment, showed low levels of anti-*Tc*CyP19 protein and negative titers of anti-*T. cruzi* antibody levels in a conventional ELISA compared to BNZnp10-treated mice, who presented higher humoral responses against *T. cruzi* and *Tc*CyP19 protein. This is in accordance with the lower levels of parasitemia, assessed by qPCR, and the less histopathological damage found in mice treated with more efficient treatments such as BNZnp50 [18].

*T. cruzi* Nicaragua isolate-infected C57BL/6J mice treated during the chronic phase of infection with an intermittent administration of BNZit75 or in combination with ALLO showed higher levels of anti-*Tc*CyP19 antibodies than those receiving continuous parasiticidal treatments. This difference has also been observed with conventional ELISA, although no differences were observed in the parasite load of both groups of experimental animals [19]. In the *Tc*N-C57BL/6J mouse model, all BNZ treatments reduced the inflammatory lesions in the heart, and ALLO addition decreased the inflammation in BNZc50 continuous treatments. No significant differences were found in anti-*Tc*CyP19 antibody levels in BNZit75-treated mice compared to untreated animals and those treated with BNZit75 + ALLO [19]. In C57BL/6J mice treated in the chronic phase of *T. cruzi* infection, continuous drug treatments showed an increased reduction in anti-*Tc*CyP19 antibodies than intermittent administration of BNZ (Figure 2B).

Trypanocidal treatments in Chagas disease have been extensively studied in adult chronic patients, being one of the main challenges in the evaluation of treatment efficiency in clinical, parasitological and serological studies.

In chronically *T. cruzi*-infected patients, it is very difficult to evaluate treatment success by measuring the conversion to negative serology in *T. cruzi* infection. Although serological tests with *T. cruzi* total proteins such as antigens are very sensitive in diagnosing an infection, they are not useful to test the success of antiparasitic treatments due to the long persistence of specific antibodies, which have been found even more than 10 years post-treatment [38,39]. Moreover, a study that followed up 430 chronic Chagas disease patients after treatment showed that a complete seronegative status was achieved in an average of 11.7 years [16].

In this context, there is a need to identify biomarkers that allow the evaluation of the treatment efficacy in a short period of time, providing information on the progression of the disease.

The quantification of parasite DNA is valuable to measure the parasiticidal treatment success in acute *T. cruzi* infections, characterized by high parasite loads in baseline samples. The measure of parasitemia by qPCR is very useful to detect the amplification of *T. cruzi* DNA as a failure of the trypanocidal treatment since it detects parasite persistence. In chronic *T. cruzi* infections, children present a larger proportion of parasite load in their pre-treatment sample [7] than adult patients. In this group of samples of *T. cruzi*-infected subjects, we were not able to detect parasitemia after treatment because most of them had no detectable parasite load in their baseline sample or a blood sample was not available. Previous studies have demonstrated that parasite hemocultures performed with blood samples from treated patients are significantly different from those performed with samples from untreated patients [39,40]. However, a negative hemoculture and negative DNA amplifications after treatment do not indicate parasitological cure, since the parasite load could be below the sensitivity of these methods or might fluctuate during the chronic phase of *T. cruzi* infection [39,41]. In this context, a multiplex serological assay has proved to be more efficient than conventional serology in evaluating subjects with chronic Chagas disease after etiological treatment, since recombinant antigens have been found to detect seroconversion at earlier time points after therapy [6].

The children and adults chronically infected by *T. cruzi* studied in this work showed significantly lower levels of antibodies against the *Tc*CyP19 recombinant protein at 12 months after etiological treatment, and this was generally sustained until 48–120 months or 30–38 months after treatment in the case of children and adults, respectively. Interestingly, we also detected a decline in the specific antibodies for *Tc*CyP19 in the group of chronically *T. cruzi*-infected children that previously had not exhibited any change in conventional serology [15]. Moreover, four out of seven of this group of children whose blood samples were analyzed by a multiplex serological assay showed a significant decline in more than two recombinant proteins at 12 months post-treatment [15].

Due to the limitations of conventional serology and the lack of reliable parasitological assays to follow up the success of trypanocidal treatments, some other *T. cruzi* molecules were assayed as potential biomarkers of an early therapeutic response. The *T. cruzi* KMP11, HSP70 and PFR2 [42]; TcTASV antigens [43]; and Tc_5171 antigen [34], among many others, have been proposed as follow-up biomarkers, reviewed in [41,44]. In particular, the *T. cruzi* Ca^2+^-binding flagellar protein F29 has been extensively studied as an early marker of response to treatment with parasiticidal drugs in samples from treated patients. A significant decrease in antibodies against the anti-F29 antigen in an in-house ELISA was noted when monitoring the response to drug treatments [45], and although a low specificity has been detected [46], recent results have shown that 77.2% of *T. cruzi*-infected children treated with a 60-day regimen with Nifurtimox seroconvert for ELISA-F29 [47]. 

Most of these recombinant antigens were recognized by sera from Chagas disease patients with statistical significance compared with the sera from healthy donors. In general, a significant decrease in the reactivity against many biomarkers was observed in a high percentage of patients soon after etiological treatment and in the reactivity during the post-treatment follow-up period.

Many efforts have been made to identify *T. cruzi* serological biomarkers, but further studies are needed regarding the assessment of therapeutic efficacy in patients living in different endemic areas and those with pathological alterations and of its specificity respect to other infectious diseases. Although the ELISA-*Tc*CyP19 described in this work requires analytical and clinical validation, this recombinant protein seems a promising tool to assess early parasiticidal treatment follow-up in *T. cruzi*-infected patients.

## 4. Materials and Methods

### 4.1. Parasites

Epimastigotes of the *T. cruzi* CL Brener clone were cultured at 28 °C in Liver Infusion Tryptose (LIT) medium (Difco) supplemented with 10% fetal bovine serum (FBS) (Natocor, Córdoba, Argentina). Cell-derived *T*. *cruzi* trypomastigotes were obtained from cell cultures by using Vero cells at 37 °C in a 5% CO_2_ atmosphere in RPMI 1640 medium (Sigma Aldrich, St. Louis, MO, USA) supplemented with 10% FBS. Axenic amastigotes were obtained by incubation in RPMI medium with 10% FBS and pH 5.0 of cell-derived trypomastigotes for 24 h at 37 °C in a 5% CO_2_ atmosphere.

### 4.2. Expression and Purification of T. cruzi CyP19 Recombinant Protein

The coding region for the TENU0559 DNA clone was cloned into a pQE30 plasmid (Qiagen, Gilden, Germany) and the *E. coli* M15 strain was transformed. *Tc*CyP19 recombinant protein expression was induced with 1 mM isopropyl-L-D-thiogalactopyranoside and purified by a Ni2-nitriloacetate agarose column, as previously described [25].

### 4.3. Obtention and Purification of Polyclonal Antibodies against TcCyP19

Specific antibodies against the purified *Tc*CyP19 recombinant protein were obtained by inoculating BALB/c mice. A mixture of 200 micrograms of *Tc*CyP19 with 100 microliters of Freund incomplete adjuvant was administered in 10 weekly doses by the intraperitoneal route. Antibodies were purified from sera from immunized mice by Protein A Sepharose^®^ High Performance chromatography (Sigma Aldrich, St. Louis, MO, USA) according to the manufacturer’s instructions.

### 4.4. Sera from Mice Infected with T. cruzi and Treated in the Acute Phase

Four-week-old female C3H/HeN mice were intraperitoneally infected with 1000 culture-derived trypomastigotes of the Nicaragua isolate of *T. cruzi* and then treated with a Benznidazole (N-benzyl-2-nitro-1-imidazole-acetamide; ^®^Abarax ELEA Lab, Buenos Aires, Argentina) nanoparticle formulation (BNZnp) for 30 days, at 2 to 32 days post-infection at a dose of 50 mg/day (BNZnp50) (*n* = 6) or 10 mg/kg/day (BNZ-np10) (*n* = 6). Control infected mice without treatment (*n* = 5) received only the drug vehicle (olive oil). Blood from uninfected mice and *T. cruzi*-infected treated and untreated mice was collected from the orbital venous sinus (500 μL) at 3 and 6 months post-infection, and serum was obtained by centrifugation of coagulated blood.

### 4.5. Sera from Mice Infected with T. cruzi and Treated in the Chronic Phase

Four-week-old female C57BL/6J mice were intraperitoneally infected with 3000 culture-derived *T. cruzi* Nicaragua isolate trypomastigotes. Mice received treatments at 3 months post-infection with continuous 30 doses of BNZ (BNZc) of 50 mg/kg/day (*n* = 5) or 75 mg/kg/day (*n* = 4), or an intermittent dose regimen of BNZ (BNZit) of 75 mg/kg/day (*n* = 9) supplemented in one dose every 7 days 13 times. In addition, in all schemes, 30 doses of 64 mg/kg/day ALLO (4-hydroxypyrazol 3, 4-d pyrimidine, Gador Lab, Buenos Aires, Argentina) were supplied, except to one group of mice that received only BNZit 75 mg/kg/day (*n* = 4). Blood from uninfected and infected treated and untreated mice was collected from the orbital venous sinus (500 μL) at 3 and 6 months post-infection, and serum was obtained by centrifugation of coagulated blood. In both sets of experiments, mice were located in a room with a controlled temperature and water and food ad libitum and then randomly selected prior to infection and assignment to the treatment groups.

### 4.6. Sera from Patients Infected with T. cruzi and Treated in the Chronic Phase

*T. cruzi*-infected adult volunteers (*n* = 16, twelve females and four males aged 22–48 years old) and *T. cruzi*-infected children (*n* = 17, eight females and nine males, 5 to 16 years old) were enrolled at the Clinical Department of INP-ANLIS Malbrán. All children were born to *T. cruzi*-infected women. Age- and sex-matched children with negative serological findings were recruited as uninfected controls. Adults and children were living in a non-endemic area (Buenos Aires) and, at the time of the recruitment, were considered infected by *T. cruzi* by our Diagnostic Department, since they were seropositive for at least two of the three tests performed: indirect immunofluorescence assay, indirect hemagglutination assay and ELISA [8]. Individuals were classified in the 0 group according to the Kuschnir clinical classification. Adult and pediatric patients were treated with BNZ, 5 mg/kg body weight per day for 60 days or with 10 mg/kg per day of nifurtimox for 60 days. One blood sample before treatment and several others after treatment were obtained from each patient, and some of them were followed up until 38 months post-treatment (Table 1). Twenty serum samples from healthy and infected but not treated adult patients who attended the Diagnostic Department of the INP-ANLIS Malbrán for diagnosis were, respectively, used as negative and positive controls for the ELISA developed to detect *Tc*Cy19 and Western blotting. Age- and sex-matched children with negative serological findings were recruited as uninfected controls.

### 4.7. Immuno-Enzymatic Analysis of Proteins Electrotransferred to Nitrocellulose Membranes (Western Blot)

The *T. cruzi* recombinant protein *Tc*CyP19 was separated by SDS-PAGE and electrotransferred from polyacrylamide gels onto nitrocellulose membranes in Tris 25 mM, glycine 192 mM and 20% *v*/*v* of methanol in Mini Protean II (Bio Rad, Hercules, CA, USA) equipment at 30 V overnight at 4 °C. Strips were blocked in 5% skimmed milk in PBS at room temperature (RT) for 1 h and then incubated at RT for 1 h with polyclonal mouse anti-*Tc*CyP19 (1:2000). For the detection of *Tc*CyP19 in the sera from mice and humans, strips were incubated for 1 h at RT with sera from anti-*Tc*CyP19 elicited in mice (1:1000), an uninfected human, a chronic adult *T. cruzi-*infected patient, an uninfected mouse, and a chronic and acute *T. cruzi-*infected mouse, all diluted to 1:100. Membranes were washed with PBS-Tween20 and then incubated at RT for 1 h with biotinylated anti-mouse IgG (Jackson, West Grove, PA, USA) (1:2000) or goat anti-human Horseradish Peroxidase (Invitrogen, Waltham, MA, USA) (1:5000) (Abcam, Cambridge, United Kingdom). After washing, membranes were incubated with streptavidin-horseradish peroxidase (Jackson) (1:1000) at RT for 30 min. Detection was performed with alpha-chloronaphtol.

### 4.8. In-House ELISA for Evaluation of Anti-TcCyP19 Antibodies (ELISA-TcCyP19)

Each well of the ELISA plate was primed overnight at 4 °C with *Tc*Cyp19 recombinant protein (50 ng/well). Subsequently, wells were washed with PBS-0.05% Tween 20 and incubated with blocking solution (PBS supplemented with 5% skim milk). After three washes with PBS-0.05% Tween, a 1:200 dilution of both mouse and human samples, controls and trypanocidal drug-treated samples were added and incubated at 37 °C for 1 h. After three washes, HRP goat anti-human IgG antibody (Invitrogen) or anti-mouse antibodies labeled with biotin (Invitrogen) and then peroxidase (Roche, Basel, Switzerland) were incubated at 37 °C for 1 h. After three washes with PBS-0.05% Tween 20, the reaction was developed using o-phenylenediamine dihydrochloride and hydrogen peroxide (0.02%) at 37 °C for 10 min. The enzymatic reaction was stopped by adding 2N H_2_SO_4_. Optical densities (OD) were read at 490 nm with an ELISA microplate reader (MINDRAY ME-96A). The results are presented as normalized signal-to-cutoff (S/Co) ratios calculated by dividing the OD value of the sample being tested by the OD value of an internal cutoff.

### 4.9. Statistical Analysis

Data analyses were performed using the GraphPad PRISM 8.0.1 software. The normal distribution of data was verified by the Shapiro–Wilk normality test. Treatment groups were compared using the Kruskal–Wallis test, followed by Dunn’s multiple comparison tests. For data with a gaussian distribution, a Paired *t*-test was applied to analyze differences in S/Co, and two-tailed *p* values were calculated. A unpaired nonparametric Mann–Whitney test was performed to compare the S/CO values of two groups. Differences were considered to be statistically significant when *p* < 0.05.

## 5. Conclusions

The use of recombinant *Tc*CyP19 as an antigen in an ELISA allowed the detection of an efficient trypanocidal treatment in mice with chronic and acute *T. cruzi* infections. The reactivity with the *Tc*CyP19 antigen also significantly decreased in the sera from chronically *T. cruzi*-infected adults and children treated with trypanocidal drugs at 12 months after treatment, while conventional serology remains reactive for decades. Remarkably, this recombinant protein could detect lower levels of specific antibodies in those treated children who sustained their conventional serological response. These results encourage us to further evaluate this recombinant protein in an increased number of *T. cruzi*-infected treated patients for analytical and clinical validation to support the use of *Tc*CyP19 as a biomarker of the efficacy of antiparasitic treatment in patients chronically infected with *T. cruzi*.

## Figures and Tables

**Table 1:** Patients included in this study.

		N	Sex (%)		Treatment		Mean Age in Years (Range)	Mean Time (Months) of Sampling after Treatment (Range)
			Female	Male	BZN (%)	Nifurtimox (%)		
Adults		16	12/16 (75)	4/16 (25)	15/16 (94)	1/16 (6)	38.9 (22–48)	11.9 (2–39)
Children	Group A	10	5/10 (50)	5/10 (50)	9/10 (90)	1/10 (10)	8.3 (6–14)	73 (48–120)
	Group B	7	3/7 (43)	4/7 (57)	6/7 (86)	1/7 (14)	12.6 (1116)	59.7 (48–73)

## Data Availability

Data are available on request to the corresponding author.
